# An adolescent treated with rapid maxillary expansion presenting with strabismus: a case report 

**DOI:** 10.1186/1752-1947-7-222

**Published:** 2013-08-23

**Authors:** Annalisa Monaco, Michele Tepedino, Lelio Sabetti, Ambra Petrucci, Fabrizio Sgolastra

**Affiliations:** 1 Life Health and Environmental Science Department, School of Dentistry, University of L’Aquila, L’Aquila, Italy; 2 Department of Clinical Applied Sciences and Biotechnology, School of Medicine, University of L'Aquila, L'Aquila, Italy; 3 School of Dentistry, Dental Clinic, University of L’Aquila, Via Vetoio 1, Building Delta 6, 67100, L’Aquila, Italy

**Keywords:** Case report, Maxillary expansion, Orthodontics, Strabismus, Vision defect

## Abstract

**Introduction:**

Few *in vivo* studies have investigated the effect of maxillary expansion on strabismus; however, some *in vitro* studies hypothesized that changes in the palatal width obtained with rapid maxillary expansion appliances could involve other bone structures that contain blood vessels and nerves conveying to the orbital cavity. The present case report seems to support that hypothesis, even if no analysis of pathogenetic mechanisms could be drawn.

**Case presentation:**

We present the case of a 14-year-old Caucasian girl affected by strabismus and referred for the treatment of a class III malocclusion with transverse maxillary deficiency, which was corrected by the application of a rapid maxillary expansion appliance (Haas type). At 2 months follow-up, the patient, who had not undergone any ophthalmologic treatment, was submitted to an ophthalmologic examination that revealed a marked change in the vision defect, which slightly relapsed at 6 months.

**Conclusions:**

The results of our clinical evaluation showed a remarkable modification of the oculomotor system of our patient as an outcome of the rapid maxillary expansion.

Further studies are needed to clarify these findings and to investigate the clinical implications of these observations.

## Introduction

There are many relationships between the oculomotor and the stomatognathic apparatus. The oculomotor system comes from the occipital somites together with the tongue’s muscles and the suboccipital muscles [1]. These structures are functionally bound and cooperate to manage the head and neck position [2]. The trigeminal system represents the connection between these somitic structures and those derived from the branchial arches, collecting the proprioception from both somitic structures and oculomotor muscles [3]. The trigeminal system is also a part of the “via oculo-cefalogira” (oculo-trigemino-cefalogira) [3], contributing to the neuromyofascial regulation of the craniocervical-mandibular rest position. Many studies have assessed the presence of connections between the trigeminal and vestibular nuclei [4] and the superior colliculus [5].

We present the case of a 14-year-old Caucasian girl affected by strabismus and referred for the treatment of a class III malocclusion with transverse maxillary deficiency, which was corrected by the application of a rapid maxillary expansion (RME) appliance (Haas type).

## Case presentation

**Figure 1:**
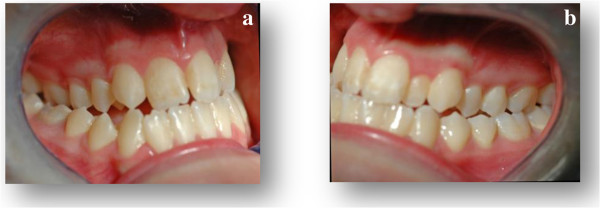
(1a, 1b) Intra-oral pre-expansion photographs.

**Figure 2:**
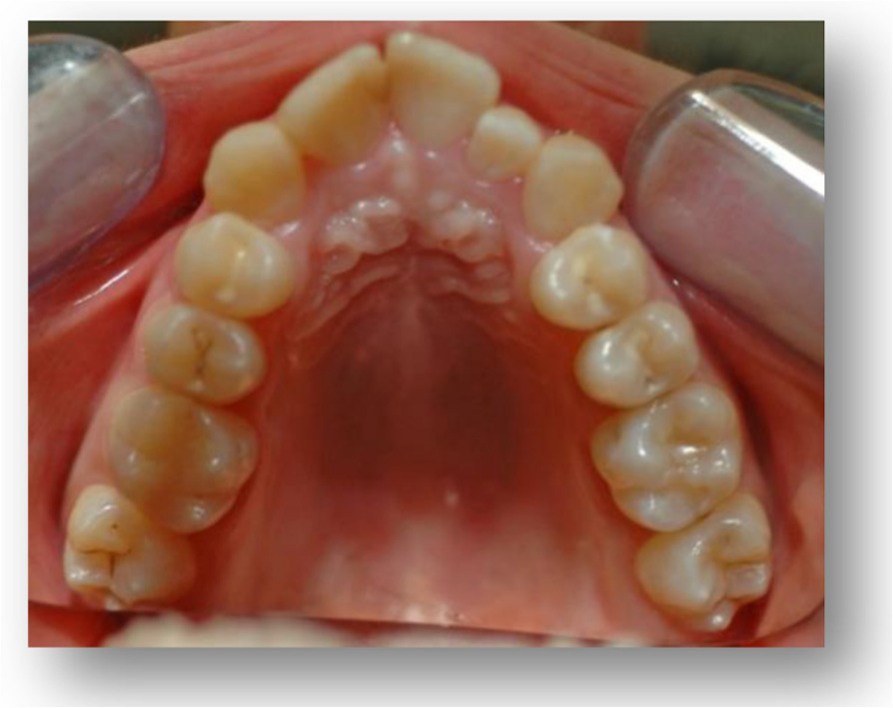
Occlusal pre-expansion photograph.

In 2010, a 14-year-old Caucasian girl was referred to undergo an orthodontic examination. Her medical history, clinical and radiographic examinations excluded the presence of systemic diseases or congenital craniofacial syndromes. However, during the collection of the anamnestic data, she reported a recent ophthalmologic examination that showed a hyperopic astigmatism in her right eye and a mixed astigmatism in her left eye. Furthermore, the orthodontic examination revealed the presence of a class III malocclusion with transverse maxillary deficiency (Figures 1 and 2).

**Figure 3:**
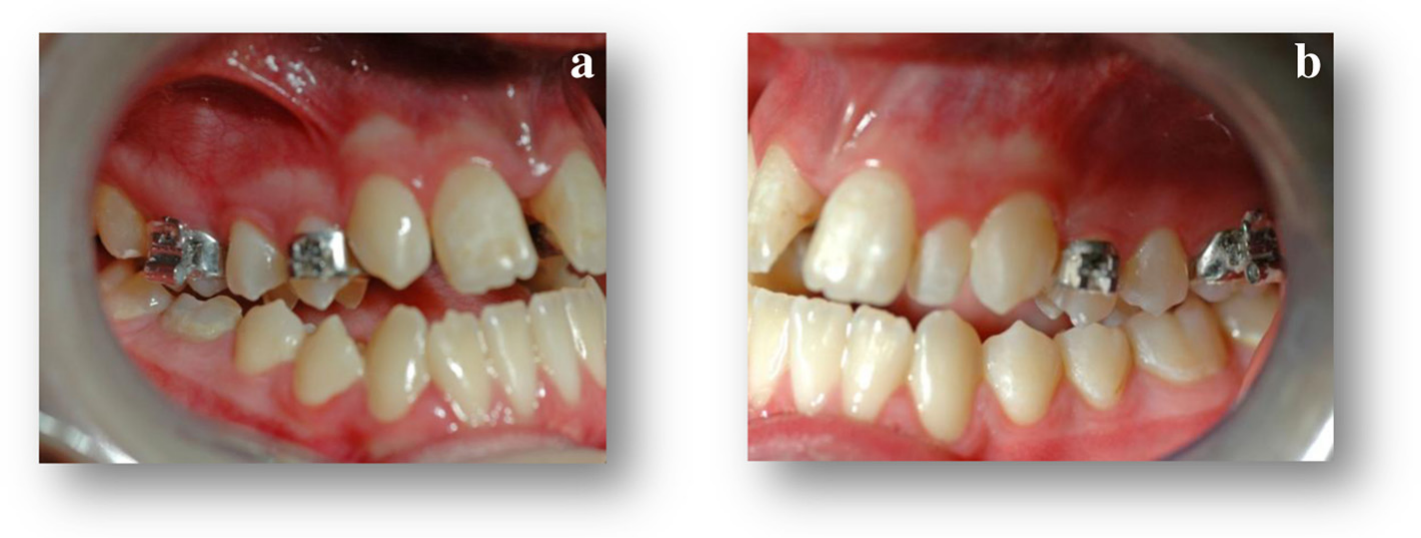
(3a, 3b) Intra-oral post-expansion photographs.

**Figure 4:**
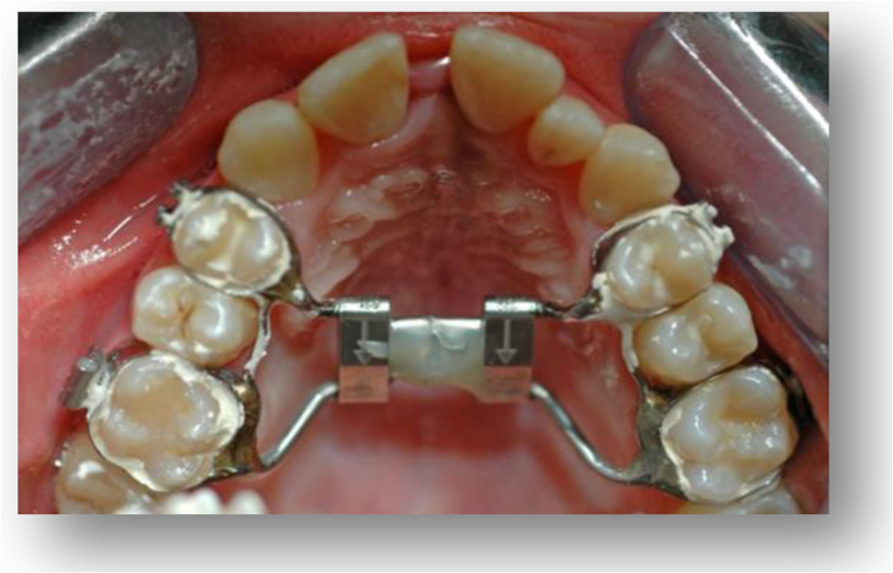
Occlusal photograph after expansion with rapid maxillary expansion appliance.

According to the guidelines proposed by Haas [6], she was treated by RME with the Haas appliance as a part of her comprehensive orthodontic treatment. For this purpose we used the Haas-type expander, with four bands (first permanent molars and first premolars) and lingual stainless steel bars of 1.0mm diameter. The appliance screw was activated two quarter turns (equivalent to a 0.2mm expansion/turn) twice a day until the expansion screw reached 6mm. The appliance was left *in situ* as a passive retainer for 3 months (Figures 3 and 4), after which it was removed.

One week before the application of the Haas-type device, the patient underwent an ophthalmologic and orthoptic examination, which showed a normal visual acuity, a hyperopic astigmatism in her right eye, a mixed astigmatism in her left eye and a normal fundus. The cover test (CT) showed an exophoria-tropia, a latent intermittent strabismus which occasionally became evident, with a V pattern deviation, where the angle of deviation became smaller going from upper to lower gaze positions. The evaluation of the extraocular muscles (MOE) function revealed the presence of a hyperfunction of lateral rectus and inferior oblique muscles in both eyes, as well as a convergence defect, with a near point of convergence (NPC) of 8cm. On the basis of those clinical outcomes, the ophthalmologist prescribed corrective glasses that the patient refused to wear.

Two months after palatal expansion, the patient came back to the ophthalmologist, who assessed a remarkable change in her oculomotor defect. The exophoria-tropia turned into a simple exophoria, since the clinical evidence of the ocular disorder disappeared; the angles of deviation were slightly improved, as well as the prismatic convergence and the NPC. The MOE evaluation showed a normal muscular function, Lang II stereotest and Worth 4-dot test revealed a good binocular cooperation. During the entire period of the orthodontic treatment she had not undergone any other medical treatments and did not report the consumption of any drug.

The patient continued her orthodontic treatment, and after 6 months we requested an ophthalmological follow-up, which showed a relapse tendency of the measured angles of deviation and convergence. However, a CT assessed the presence of a simple exophoria, confirming the improvement of her ocular defect at 6 months.

An ophthalmologic examination was performed according to the guidelines of the American Optometric Association [7] and included the visual acuity assessment, both in natural and cycloplegic (tropicamide 1%) position, funduscopic examination, alternate CT, Berens prism test, NPC, fusional convergence, Lang II stereotest, Worth 4-dot test and the evaluation of MOE function. The alternate CT is used to detect the presence of evident (heterotropia) or latent (heterophoria) ocular deviation: the patient is asked to fix a straight ahead target, at distance, near and in different gaze positions, while the examiner occludes one of the patient’s eyes with an opaque card or paddle; by observing the uncovered eye as the cover is placed over its fellow eye, the operator looks for a loss of bifixation. The Berens prism test uses a source of light produced with a calibrated lamp and Berens prismatic bars (that appear as a series of prisms of growing strength, joined side to side); the incident ray, emitted from the object, is deviated toward the base of the prism so that the image of the object is exteriorized toward the top of the prism. The diagnostic examination consists of the measurement of the angle of deviation and then in finding the angle obtained from the two visual axes. The NPC test is used to record the distance at which fusion is lost and one eye diverges; it uses a luminous stick which is drawn slowly through both eye planes to the base of the nose, while the patient is asked to maintain fixation on it.

The Lang II stereotest consists of a card (9.5×14.5cm) that contains pictures of three items, which represent disparities of 600, 400, and 200 seconds of arc respectively, at a 40cm-test distance. A control image is visible monocularly. To evaluate stereopsis the patients are asked whether they can see any picture on the card. The Worth 4-dot test consists of a modified flashlight with four holes, approximately 1cm in diameter, organized in a diamond shape, usually arranged with the top hole showing only red light, the left and right holes showing only green light, and the bottom hole showing white light. The patient wears anaglyphic glasses (with one red lens over one eye, usually the right, and one green lens over the other eye, usually the left) and is asked to fix the lights. Because the red filter blocks the green light and the green filter blocks the red light, it is possible to determine if the patient uses both eyes simultaneously in a coordinated manner. The evaluation of MOE function is performed asking the patient to fix an object in nine gaze positions; it assesses the normality, the deficiency or the excess of eye movements, as well as the presence of nystagmus.

All evaluations were performed by the same experienced examiner.

## Discussion

From a clinical point of view, many authors have studied the effects of dental occlusion and tongue position on vision. Sharifi Milani *et al.*[8] described the effects of occlusal modification caused by a mandibular orthopedic repositioning appliance on visual focusing. Deodato *et al.*[9] obtained the same result modifying the tongue’s posture: they observed the resolution of the oculomotor disorder in a pediatric patient affected by asthenopia, by altering the tongue’s position with the application of roundish composite shims over the lingual face of the superior incisors. Many studies conducted by Monaco *et al.*[10-15] showed the presence of a relationship between malocclusions, temporomandibular disorders and visual defects, remarking a higher prevalence of myopia in patients with class II malocclusions than in patients with class I and III malocclusions, as well as a higher prevalence of patients with astigmatism and cross-bite.

Many studies based on histologic methods, radiologic imaging, photoelastic models, bone scintigraphy and finite element analysis hypothesized that the forces derived from palatal expansion could spread to deeper anatomical structures. Circumaxillary sutures, as well as other structures not directly joined with the maxillary bones, were affected by this strong orthopedic force, showing different levels of stresses, displacement and an increased cellular activity. In agreement with those observations Lanigan and Mintz [16] reported the case of a fracture of the cranial base following palatal expansion, and Gardner and Kronman [17] observed the opening of the spheno-occipital synchondrosis in their study on rhesus monkeys as a consequence of palatal expansion.

Through finite element method analysis, which has been widely proved to be a reliable and effective method, several studies quantified the bone displacement and the stress distribution pattern derived from palatal expansion. High stresses were recorded in the zygomatic bone and mostly in the sphenoid bone, as well in the base of the pterygoid plates, in the superior orbital fissure, in the round foramen, in the oval foramen, in the spinous foramen and in the optic foramen [18-20]. Recently, Sicurezza *et al.*[21] analyzed the changes in the volume and aperture width of the ocular cavity as a consequence of palatal expansion, through cone-beam computed tomography evaluation. They found that the orbital volume increased from 18.81 ±1.23mL to 19.53±1.26mL and that the anterior width of the ocular cavity (measured from the posterior lacrimal crest to the frontozygomatic suture) increased from 36.02 ±1.24mm to 37.11 ±1.01mm.

## Conclusions

In conclusion, it could be hypothesized that the changes in the palatal width obtained with RME appliances can involve other bone structures that contain blood vessels and nerves that convey to the orbital cavity; however, further studies are needed to clarify these findings and to investigate the clinical implications of these observations.

As stated above, we can try to understand the results of our clinical evaluation: in this case, we observed a remarkable modification of the oculomotor system of the aforementioned patient as an outcome of the RME.

## Consent

Written informed consent was obtained from the patient's parent for publication of this case report and accompanying images. A copy of the written consent is available for review by the Editor-in-Chief of this journal.

## Abbreviations

CT: Cover test; MOE: Extraocular muscles; NPC: Near point of convergence; RME: Rapid maxillary expansion.

## Competing interests

The authors declare that they have no competing interests.

## Authors’ contributions

The patient was under the care of AM; AM operated on the patient. AP and FS analyzed and interpreted the data. MT wrote the manuscript. LS made additions to the manuscript. All authors reviewed and approved the final manuscript.
